# Marfan Syndrome in an Iranian Family: A Case Series

**Published:** 2014-07

**Authors:** Mohammad Hossein Davari, Toba Kazemi

**Affiliations:** Birjand Atherosclerosis and Coronary Artery Research Centre, Birjand University of Medical Sciences, Birjand, Iran

**Keywords:** Marfan syndrome, Eye Manifestations, Ectopia Lentis

## Abstract

Marfan syndrome (MFS) is a genetic disorder which is inherited by autosomal dominant traits. In MFS, lens displacement and cardiovascular involvement are important causes of morbidity and mortality in the clinical course of the disease. In this case study, the ocular involvement in a family with severe penetration of MFS is reported. Twelve members of a family (father, two daughters, three sons, and six grandchildren) had MFS. Lens ectopia was the most common ophthalmic involvement among the family (100%). Other ocular involvements were as follows; Hypoplastic iris or ciliary’s muscle hypoplasia (50%), on gated eyeball (42%), flat cornea (30%), glaucoma and cataract (25%), retinal detachment (16%). Three members of the family underwent eye surgery including lens extraction, glaucoma surgery and retinal surgery.

## Introduction

Marfan syndrome (MFS) is a connective tissue disorder being transmitted as an autosomal dominant.^1^ The cause of MFS is molecular defect in fibrillin gene on chromosome 15 and it can affect several organs, including the eyes, heart, skeleton, and blood vessels. Commonly, 75% of MFS patients have affected parents.^2^ Ocular features are: 70 to 80% of patients with MFS have ectopia lentis which is the most common form of ocular involvement.^3^ Eye involvement is seen in Marfan syndrome in other forms such as; abnormal flat cornea, increased eyeball axis, hypoplasia of ciliary iris muscles, glaucoma, cataract and retinal detachment. Ocular involvement in MFS is probably due to the fibrillin in the capsule of the lens, iris, ciliary body, and sclera.

Eye involvement in the syndrome occurs in two forms; namely major and minor, where ectopia lentis is in the major category. For an eye to be involved at least two minor criteria amongst the abnormal flat cornea, increase in the eyeball’s axis and hypoplasia of ciliary iris muscles must be met.^4^ Generally, 8%-50% of patients with MFS have retinal detachment, but this occurs in about 75% of patients under the age of 20 year olds. The prevalence of myopia in MFS increases with age to the extent that it is rare among individuals less than three years old. Structural abnormalities in the iris result in weakened and dilated pupils.^3^

In this paper, the ocular manifestation of MFS in a family located in Birjand (East of Iran) is reported. Previously, the cardiovascular manifestation of this family was reported.^5^

## Case Report

Initially in 1998, a 17-year-old young man (second son of a family) was referred to an ophthalmologist for the evaluation of low vision. Ophthalmic examination of the patient revealed that the patient had iridodenosis and lens displacement. Other signs including tall stature and arachnodactily created suspicion of MFS and the patient was then referred to a cardiologist for further investigation. Given the mitral valve prolapse (MVP) and aortic root dilation, MFS diagnosis was established.

All members of this family underwent complete eye examination using the slit lamp, autorefractometer, retinoscope, ophthalmoscope, glasses box, tonometry and complete cardiac test. Through clinical indicators MFS was diagnosed and was confirmed by genetic and molecular tests with high level of certainty. In the past, the main diagnostic criteria was the Ghent’s nosology^6^ but according to a new diagnostic criteria as delineated by an expert group in 2010, mutations in the FBN1 gene only differentiate Marfan syndrome from the other aortic syndromes.^6^^,^^7^ With the cooperation of the Avicenna Research Institute (Mashhad University of medical Sciences), Marfan syndrome was confirmed genetically by DNA extraction and linkage analysis of 8 STR markers *of FBN1*.^8^

**Figure 1:**
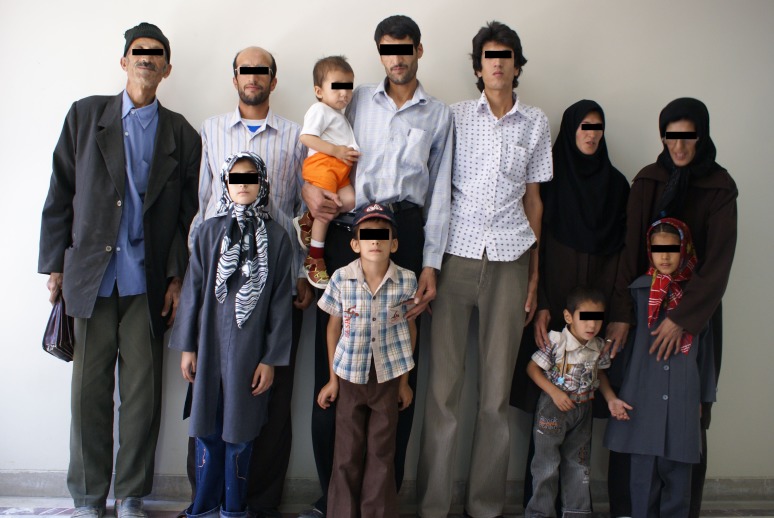
Family members with Marfan’s syndrome. From left to right: father, first son and his daughter, second son and his two sons, third son, second daughter and her son, first daughter and her daughter.

All members of this family had been periodically visited by a cardiologist and an ophthalmologist. In total, there were twelve involved individuals (the father, his three sons and two daughters as well as six grandchildren) with MFS in this family. It is the authors’ belief that MFS with such severe genetic penetration is rare.^9^ A photograph of the family under discussion is presented in figure 1. As shown in table 1, statistics for the ocular manifestation in that family are; lens ectopia 100% (12/12), flat cornea 30% (4/12), on gated eyeball 42% (5/12), hypo plastic iris or ciliary’s muscle hypoplasia 50% (6/12), glaucoma  and cataract 25% (3/12),  retinal detachment  16%(2/12). Among the family, three patients required ocular surgery.

**Table 1:** Ocular involvement, complication and treatment of the family members with Marfan Syndrome

**Case**	**Ocular involvement**	**Complication**	**Treatment**
Father	Iridodonesis, Ectopia lentis, Flat cornea, CMH BCVA=0.1, IOP=normal	Cataract	Lens extraction os, follow up annually
1st daughter	Iridgdonesis, Flat cornea Ectopia lentis, CMH BCVA=0.2, IOP=30	Two pregnancy without complication	Lensectomy+vitrectomy+bukeling+silicon oil+glaucoma surgery, F/U annually
2nd daughter	Iridodonesis, Ectopia lentis, CMH BCVA=0.3, IOP=normal Blindness OS	one pregnancy without complication, Aortic surgery	Glasses, Follow up annually, Propranolol, warfarin used due to Aortic surgery
1st son	Iridodonesis, Ectopia lentis, CMH BCVA ou=0.4, IOP=normal	Surgery for cataract &RD, Aortic surgery	Glasses, follow up annually, Propranolol, warfarin used due to Aortic surgery
2nd son	Iridodonesis, Flat cornea Ectopia lentis, BCVA, OD=4m CF BCVA, OS=0.4, IOP=normal	Surgery for RD, Aortic surgery, Amblyopic OD	Lensectomy+vitrectomy+bukeling+silicon oil, Glasses, F/U annually, Propranolol, warfarin used due to Aortic surgery
3rd son	Iridodonesis, Ectopia lentis, BCVA, OD=0.1 BCVA, OS=0.5, IOP=normal	Aortic surgery Amblyopic OD	Glasses, Follow up annually, Propranolol, warfarin used due to Aortic surgery
1st grandchild	Iridodonesis, Flat cornea,Ectopia lentis, BCVA, OD=0.2 BCVA, OS=0.5, IOP=normal	Amblyopic OD	Glasses, Follow up annually
2nd grandchild	Iridodonesis, Ectopia lentis, CMH BCVA, OD=0.5 BCVA, OS=0.5, IOP=normal	No	Follow up annually
3rd grandchild	Iridodonesis, Ectopia lentis, BCVA, OD=0.6 BCVA, OS=0.3, IOP=normal	Amblyopic OS	Follow up annually
4th grandchild	Iridodoneqis, Ectopia lentis, CMH BCVA, OU=0.5, IOP=normal	No	Follow up annually
5th grandchild	Iridodonesis, Ectopia lentis, BCVA, OU=0.5, IOP=normal	no	Follow up annually
6th grandchild	Iridodonesis, Ectopia lentis, IOP=normal	No	Follow up annually

BCVA=Best correct visual acuity; CF=Count finger; RD=Retinal detachment; CMH=Ciliary’s muscle hypoplasia; IOP=Intraocular pressure

Patient No-1 (father): OD=Lens extraction, OS=lens extraction due to senile cataract.

Patient No-2 (first daughter): OD=lensectomy+RD surgery+glaucoma surgery.

Patient No-3(second son): OD=lensectomy+IOL, OS=lensectomy+IOL+retinal surgery. 

**Figure 2:**
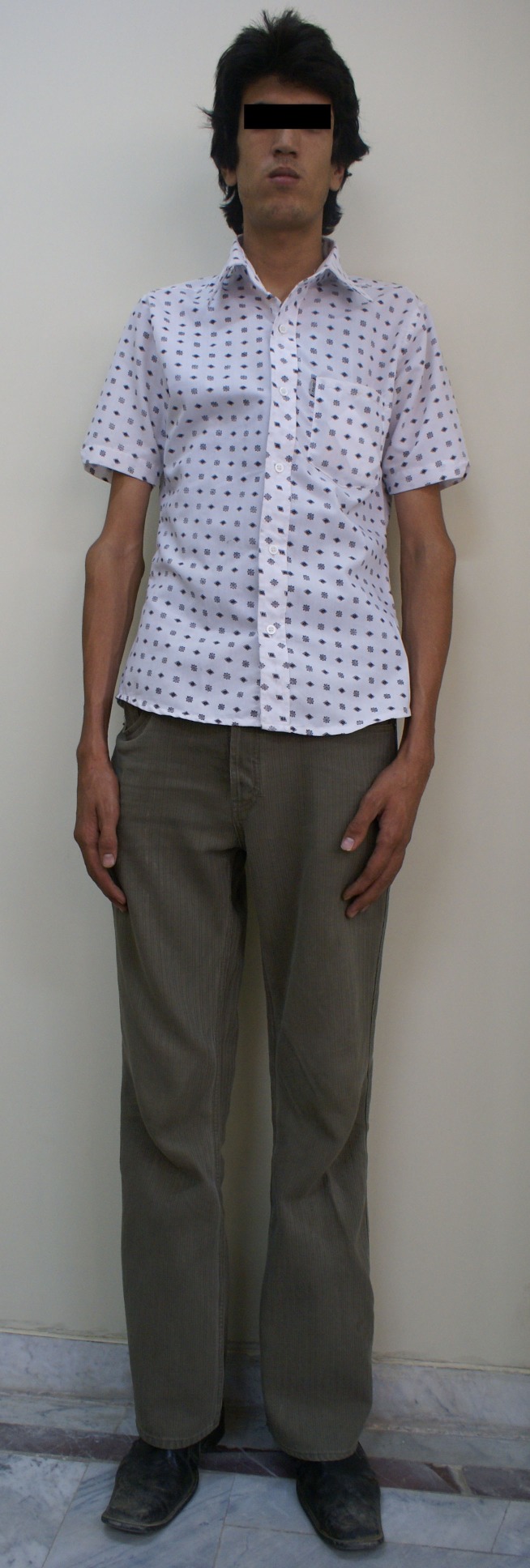
Musculoskeletal manifestation of MFS in our patients,Long Stature.

**Figure 3:**
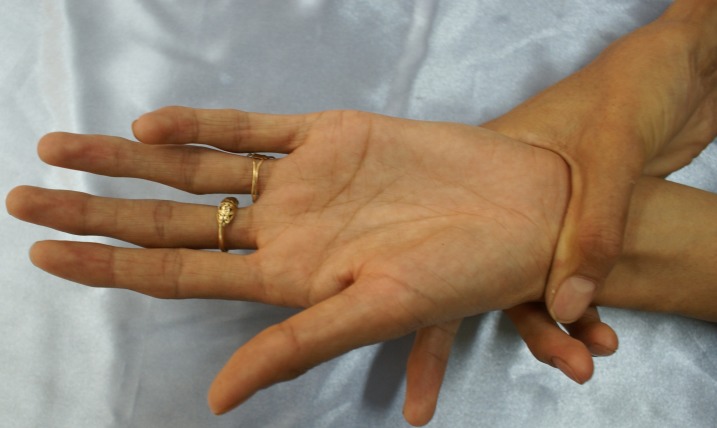
Arachnodactyli :musculoskeletal feature of MFS.

**Figure 4:**
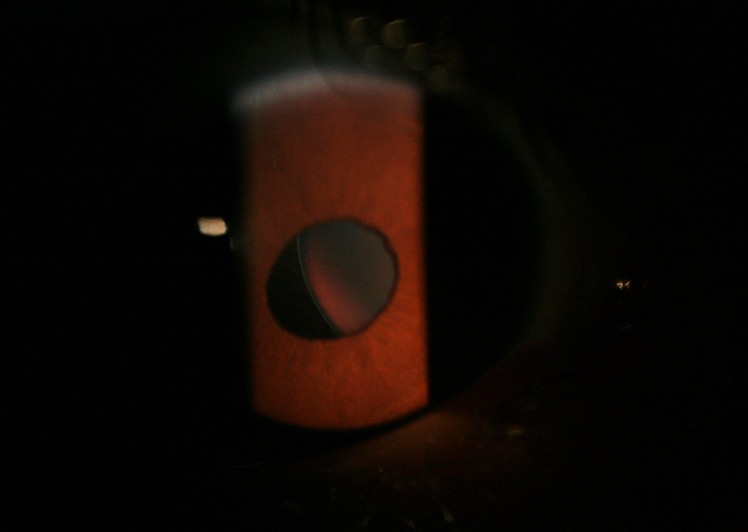
Lense dislocation in MFS.

The musculoskeletal disorders of MFS in our patients are shown in figure 2 and 3 (long stature and arachnodactyli). In figure 4, the face of one patient and the lens dislocation in an eye is shown.

## Discussion

MFS is a multisystem disease. Cardiovascular involvement (dilatation of the ascending aorta) is the most common cause of mortality in MFS patients. Ocular involvement in MFS can be serious although not fatal. In the case of the patients under discussion, the most common ophthalmic involvement in order of priority was lens ectopia, hypo plastic iris, on gated eyeball, flat cornea, glaucoma, cataract and retinal detachment. During the follow-up period, three patients required surgery due to cataract, glaucoma and retinal detachment. Vitreous involvement of the eye in patients with MFS is appropriately explained. The prevalent change is liquefaction of the gel and posterior vitreous detachment.^10^ Usually, such patients are prone to serious vitreous and retinal complications such as glaucoma and complete enlargement of retina. Consequently, in order to prevent severe decrease in vision, it is necessary for these patients to receive fast and urgent treatment.^10^

Patients with MFS should be regularly examined for refractive defect. If refractory defect occurs, it could be quickly resolved. Increased length of the globe due to myopia would increase the risk of retinal detachment. To prevent amblyopia, refractive errors should be detected and corrected as soon as possible. Correction of refractive errors is important in such patients before the age of 12 to restore visual acuity. Also in patients with MFS, intraocular pressure (IOP), lens condition (its stability and opacity) and fundus examinations should be regularly controlled for the early detection of retinal complications. To prevent retinal detachment, vitrectomy and lensectomy can be effective in improving visual acuity in some patients. The most serious signs and symptoms associated with MFS involve the cardiovascular system. An aortic dissection is often fatal and requires an emergent surgery. Four of the patients in this study underwent cardiac surgery that included aortic root and aortic valve replacement (Bental procedure). While there is no cure for Marfan syndrome, taking propranolol is recommended in preventing the development of aneurysm of aorta or its stiffness and decreasing mean arterial pressure. This medication also decreases intraocular pressure but if such pressure is felt it must be administered.

## Conclusion

Patients with MFS should be regularly monitored by a medical team that includes cardiologist, ophthalmologist and orthopedics. Cardiologist should monitor the heart valves and the aorta by echocardiography. An ophthalmologist should investigate the IOP, lens, retina and optic nerve annually. Patients with MFS should immediately consult their doctors if any heart or ocular symptoms such as chest pain, dyspnea, floaters and glare in the visual field are experienced.
